# Hospital and Institutional News

**Published:** 1920-01-17

**Authors:** 


### HOSPITAL AND INSTITUTIONAL NEWS.

### THE LATE SIR WILLIAM OSLER.

On another page (page 361) we give a sympathetic study of Sir William Osler at Oxford. His was ever a great, moving, life-giving personality, and those who care to realise what he meant in his student and graduate days to Canada as Professor of the Institute of Medicine, McGill University, Montreal, or to the United States as Professor of Clinical Medicine at the University of Pennsylvania, Philadelphia, or of the life-giving inspiration and abiding influence which he left behind him on vacating the professorship of medicine to the Johns Hopkins University, Baltimore, will be able to apprehend something of what Osler was and what he accomplished in these earlier days on referring to page 409 of The Hospital of July 19, 1919. Readers of The Hospital may like to have their memory jogged by recalling Sir William Osler's Presidency of the British Hospitals' Association Congress, held at Oxford in 1913. Osler told the writer that he had never before had an opportunity of attending a congress consisting of men who were interested mainly in the administrative side of hospital life and the important aspects and interests connected with this branch of work. He attended every sitting of the Congress and occupied the chair. He never missed a single paper or discussion, and explained that the whole experience was one of complete novelty, which gave him an insight into a side of hospital life that he had never realised. He rejoiced that it had been possible to organise such an Association and bring together so many men, the majority of whom were young men, who were keen on their work and ambitious to make it helpful and worthy of the grave responsibilities which devolved upon the treasurers, chairmen, superintendents, and secretaries of great voluntary hospitals in this country. We do not think it is too much to declare that he formed a warm and lasting affection for the Association and those connected with it. He assured us that it was his intention to attend subsequent congresses because he was certain that he would there learn many things that he ought to know, not the least being the atmosphere of reality and earnestness which characterised the utterances and methods of a large number of the members, whom he had the pleasure of meeting in the course of the proceedings in the city of Oxford.

### THE LIFE WORK OF THE MAN.

Many biographies of Osler have been written, and the medical press has been full of articles dealing with the man, and his work, but having known Osler for over forty years and loving him as we believe he loved us with a very real affection and sympathy, as co-workers in great fields of progress, having momentous issues for the people of all classes and both sexes, we are confident that his life's work was to take with him everywhere an atmosphere of trustful, keenly observant, impartial, outspoken, uplifting, purposeful endeavour to learn what there was to learn, everywhere, and to give of his best to all with whom he came in contact, pointing and living up to the highest standards in all things, which his many-sided character, great industry, powers of work, and continuous fight for righteousness, brought home to him, forming, as these did, the bedrock of existence and the mainspring of his life's action. It was delightful to meet him everywhere and always. The interval of years between one meeting and another made not the slightest difference, for whenever he and an old friend met they were at once like boys together, trustful, uplifted, joyous, merry-hearted, full of humour, which not only made the time pass and become memorable in after life, but attracted to the meeting-place, whenever possible, the best minds and men who knew of the meeting. These were bent on sharing the atmosphere and being raised out of ordinary daily routine to a level where spirituality and the highest ideals formed the basis and continuous inspiration of all who were fortunate enough to assemble on these occasions.

### FINANCING THE VOLUNTARY HOSPITALS.

Week by week we are receiving new and important evidence of the initiation of fresh efforts and the successful organisation of means, whereby the finances of our voluntary hospitals throughout Great Britain are being materially increased, so that the progress made towards financial security is almost everywhere advancing month by month. At the annual meeting of the contributors to the Royal Infirmary, Edinburgh, on the 5th instant, it was announced that the managers are about to issue a direct appeal for a large sum to meet necessary capital outlays, and at the same time to provide for the necessary augmentation of the annual revenue. Coming, as the announcement does, immediately after the inspiriting articles in the *Glasgow Herald*, from which we anticipate important financial results, we are hopeful that Scotland will run a neck-and-neck race with England and Wales in their efforts, adequately, to finance the voluntary hospitals throughout these countries by June 30 next. In the case of Scotland, we should like to hear that the St. Andrew's Red Cross organisation is alive to the movement announced by Sir Arthur Stanley in regard to the hospitals of the Metropolis and throughout England and Wales. In this case Scotland will have cause, like ourselves, to bless the Red Cross organisation, and its people to resolve to contribute continuously to its funds and to continue the personal service, or much of it, which they rendered through the Red Cross with such invaluable results during the war.

### HOW CO-OPERATION MAY BE RENDERED.

We have asked elsewhere for a direct intimation from the hospital authorities, whose needs and position were set forth with some fulness in our issue of December 20, pages 267-276 inclusive. The meeting of the British Hospitals' Association on the 23rd instant should mark a new departure, by an exhibition of great and continuous energy on the part of every reputable voluntary hospital throughout Great Britain, from that date onwards to June 30 next. We shall want an army of voluntary workers in each county, willing to render personal service in the district where they may reside. In this way, alone, will it be possible, with the co-operation of Red Cross workers throughout the country, to canvass and bring the facts of the position to the notice of the 50 per cent. of the men and women resident throughout England, Wales, and Scotland, who have not heretofore contributed anything to the support of the voluntary hospitals. These 50 per cent. have not that intimate knowledge of the work and succour which these institutions continuously render for the protection of residents in Great Britain. Offers of service, information of the appointment of a Special Committee, or the adoption of a resolution, as at Edinburgh, to organise direct appeals and a thorough canvass in every district of each county, will be welcome, and should be sent to the Editor, THE HOSPITAL, 28/29 Southampton Street, Strand, London, W.C. 2, whenever and wherever these co-operating movements may be started, from the date of the present issue onwards. Things are moving very quickly in the present day, and the outlook for the Future Sustenance and Extension of the Voluntary Hospitals has so changed for the better and improved since we began our crusade of hope, energy, and resolute propaganda, that we have reason to thank God and take courage. For we believe that June 30, 1919, will be memorable for all time in the history of the voluntary hospitals by showing magnificent results in the direction of financial improvement. These results will place these hospitals upon a new and sound basis and secure their continuance, development and greatly increased usefulness and call to all classes of the people to be up and doing in efficiency. Wherever such an institution is to be found, working for the protection and health preservation of the English, Scottish, and Welsh inhabitants of the United Kingdom. There is the urgent call to all classes of the people to be up and doing in the cause of the Voluntary Hospitals everywhere.

### THE LIFE OF THE VOLUNTARY SYSTEM.

IT gave us very real pleasure to receive evidence of the continuous and able efforts of Mr. Arthur Griffiths, of the East Suffolk Hospital, one of the most enterprising and successful organisers connected with the voluntary system in Great Britain. He is connected with a centre of hospital activity which is a characteristic representative of the life of the voluntary system. We give on another page an account of work done and important developments which have taken place there. We are reminded that we made a full and detailed inspection of the East Suffolk and Ipswich Hospital some years ago, and took some pains to give what help we could to the administration of that institution and those responsible for it. We congratulate and thank those responsible upon the splendid efforts they have put forward and the remarkable success with which they have been attended. It will be seen from the report published that the hospital now possesses a staff of physicians and surgeons, who constitute the present honorary staff. Further, six years ago the hospital urgently needed an increase of income amounting to £3,000 a year, in order that the work which lay ready to hand should be expanded and brought up to date. There was at that time an opening for the establishment of a semi-convalescent home, a branch of development in our large hospitals up and down the country which has about it the charm of the highest humanity. With the aid of these establishments it becomes possible so to arrange for the treatment and cure of the breadwinners, who may find their way, when ill, into hospital, that when they shall leave and return to their homes they may be in a condition of health and strength fitting them to commence work forthwith, and so to earn the maximum wage for their families. Such a home has been established in connection with the East Suffolk Hospital, together with the other important matters just mentioned.

### AN UPLIFTING REPORT OF STEADY PROGRESS.

Again, there was in our opinion a call to the managers of the East Suffolk and Ipswich Hospital to reconstruct a portion of the old Nurses' Home, to abolish the use of cubicles, and to secure an extension of the buildings whereby each nurse should be provided with a separate room for her use. It is, indeed, a pleasure to report that this recommendation has now been completely carried out, and when it is added to the other great changes and improvements we have just notified we make bold to say that there are probably few, if any, hospitals which can give a more uplifting report of steady progress and courageous administration, producing a maximum of good results, than that which we here have the pleasure to record in connection with the East Suffolk and Ipswich Hospital. We must now make a point of visiting this institution at an early date in order to have the pleasure of congratulating everybody concerned, and of absorbing the evidence which is here provided to the observer of what an able and live administration can secure, with the co-operation of the people for whom a great hospital exists. Such co-operative effort secures the presence throughout the institution of the right spirit, and the earnest, purposeful resolve that nothing but the best is good enough for the traditions, and the high state of development, which characterise the work that these ladies and gentlemen have the privilege of being responsible for. Instead of publishing a special article of our own this week on the Voluntary System we are publishing the account of how that system has been maintained in East Suffolk. In this way we hope to encourage hospital authorities, everywhere throughout Great Britain, to send us similar accounts of the work in every life centre up and down the country, where co-operation is active in the development of the voluntary system, with the view of making it everywhere as perfect and up-to-date as possible.

The publication of these accounts of such work will be one of the most fruitful aids to the adequate organisation of Great Britain, and the speedy and adequate financing of the voluntary hospitals.

### THE LATE SIR THOMAS FRASER.

It is with great regret that the profession has received news of the death of Sir Thomas Fraser, Emeritus Professor of Materia Medica in Edinburgh University and Honorary Physician-in-Ordinary to the King in Scotland. It was in 1877 that he succeeded Sir Robert Christison in the University appointment, and those of Scottish training will well remember his pioneer work in the teaching of a subject at that time unsatisfactory from many points of view. The student's idea was concentrated on the preparation of the drug from its crude origin, he learnt much of how to make actual pills, ointments, and the like, a training valuable in some senses, but markedly deficient in the vital matter of exactly how and when the drugs acted on the human body. The aim of Fraser's life at this time was largely to understand exactly in what way drug actions were centred, and by what means these actions might be graded to efficiency in the combat with disease. Almost may it be claimed that he opened a new era in teaching of human medicine, and for the definite fruits of his work we need but recall the valuable researches in the cardiac action of strophanthus, the ophthalmic power of physostymine, and many others. Among the many honours which fell to him it may be recalled that he was knighted in 1902, he was a Fellow of the Royal Society, President of the Royal College of Physicians of Edinburgh, Hon. LL.D. Aberdeen, Glasgow, and Edinburgh, Hon. Sc.D. Cambridge, Hon. M.D. Dublin, and Laureate of the Institute of France.

### REWARDING RESEARCH.

As seemed so obviously right and just, both to the men and the requirements of the times, the Joint Committee of the British Medical Association and the British Science Guild has published its definite recommendations that rewards for useful medical discovery should be given by the State and should take the form of pensions of from £500 to £1,000 a year. The letters of Sir Ronald Ross, a member of the Committee, to which we recently drew attention, left little doubt as to what this finding would be, and now that it has taken the form of an official recommendation the first step is satisfactorily over. The matter will be raised in the House of Commons early in the new Session, and although to some, in these times of political and financial pressure, it may appear as relatively a small and unimportant matter, we would urge, as before, that it amounts to the paying of a debt which the country would indeed do well to pay in advance. Considerable notice has been given to the recommendations now they are out, and we trust that they may similarly or even more strongly be brought before the public at the time of final Parliamentary consideration.

### DEATHS FROM FOOD-POISONING.

There have already appeared several announcements in the daily Press of mysterious deaths of possible food-poisoning origin, and this fact recalls the scare created about two years ago of a supposed outbreak of "botulism." At the same time we hear of sundry small outbreaks of suspected influenza, and in the very near future it is more than likely that we shall see the public being treated to another mystifying series of reports of unaccounted-for illness and death, and casting corresponding blame on the medical profession and research workers generally. Therefore, it is well to recall, as was very clearly explained in the last report on the health of the London public, that at the coming season there has in recent years been a definite outbreak of cases which were possibly associated as forerunners of the war-time influenza. Encephalitis lethargica, the Heine-medin disease, even cerebro-spinal fever fall into this category. They have indeed appeared before, but the terrible ending in the epidemics of 1917-1918 brought them into extra prominence. We call attention to this relation, although space does not allow of detailed explanation, in the hope that the public may not be led by scare publications unduly to fear that because we again have a few of these curious and difficult cases with us that a death-dealing epidemic is again inevitable.

### PLAIN WORDS FROM A CORONER.

On January 9 Mr. S. Ingleby Oddie held an inquest at Wandsworth on the body of a demobilised non-commissioned officer who had committed suicide by jumping in front of a moving train at Earlsfield. It appears that the young man had contracted syphilis, and this fact had so preyed on his mind that he took his own life; before doing so, he wrote a very pathetic letter to his sister in which he implored her to instruct her own children fully in those perils to which he had succumbed. The coroner said it was, doubtless, a very unpleasant matter for parents to discuss sexual matters with their sons, but though unpleasant, he agreed with the unfortunate deceased that it was the duty of parents to warn their sons of the dangers of promiscuous sexual acts. This was a case where the proverb was wrong in saying that "where ignorance was bliss it was folly to be wise." We understand that the Society for the Prevention of Venereal Disease is preparing a scheme which will provide for the correct tuition of parents as to their duties in these matter; and we are sure that such propaganda will do much to cure these venereal diseases which exist rampant among the community in this present-day epoch of moral instability.

### DISSECTING-ROOM DIFFICULTIES.

It will hardly come as a surprise to the institutional world that the anatomical departments of the London medical schools are faced with a great shortage of bodies for dissection. At the present time, owing to the large number of students and the almost unprecedented scarcity of their anatomical requirements, and almost hopeless situation has been reached in several quarters. It is perhaps true that in the past an excessive amount of time has been devoted by the beginner to pure anatomical dissection, but nevertheless, there is a great and obvious disadvantage in the fact that many students are now forced to watch procedures that, even though they may not ultimately practice as surgeons, it would be far better for them to perform for themselves, and the only hope of a solution lies in a revision of the Anatomy Act. It is, to-day, the duty of the London Anatomical Committee to obtain and distribute bodies among the medical schools, but they are restricted to direct application and simple urgent request for unclaimed bodies to the boards of guardians. The old-age pension scheme considerably reduced the number of such bodies available, and also, legally, the guardians have the option of buying them if they wish to. Therefore, the matter is one to be viewed in the broadest way, considering the urgent medical requirements of the country, and a revision of the Anatomy Act would seem to be its only satisfactory solution.

### PRINTERS WHO LEAD THE WAY.

It is gratifying evidence of the widespread and extending desire, which finds expression among the working classes, to help the hospitals, and of their genuine interest in these institutions, that the printers and employees of Spottiswoode, Ballantyne and Co., Ltd., New Street Square, during the last quarter of 1919 collected for the hospitals and sent to the Hospital Saturday Penny-a-Week collection the sum of £18 13s. 9d. We hope this example may be followed, without a single exception, throughout the printing firms and their employees in the Metropolis throughout Great Britain. If this can be done not only will the men themselves benefit directly when ill, but indirectly and continuously through the personal service and substantial help they will spontaneously render to the hospitals through their weekly contributions.

### THE MOTHERS' DEFENCE.

We read reported in a recent number of *New Witness* an instance of a "typical case of oppression by officials," the details being supplied by the Mothers' Defence League. We have previously expressed our opinion of the spirit which this body is attempting to develop among the people and the tremendous harm it may in future do. In this case the National Society for the Prevention of Cruelty to Children is attacked, or more correctly the action of one of its officials. Inasmuch as the inspector had taken the children away without a magistrate's order, it was obviously easy legally to prove that the children were improperly taken from their mother. So far so good, but the discovery of this fact and the "score" of the League over the Society, reflects, as far as we can see, no particular credit either on the motives or the ideals of the former. The more so in that they publish an account of the affair, ending, "The act is illegal, and in the interests of public justice it is to be hoped that an action for trespass will be brought against the Society," but entirely omit to give any indication of the inspector's reason for considering the children best removed from their mother's care. At least we know that two children aged seven and fifteen were left alone in the house while the mother was at work. For how long? Under what conditions? These are other facts which the public should also judge and know.

### FUTURE OF DUBLIN HOSPITALS

In a recent address on the future of Dublin hospitals, Dr. E. M. Fannin said that there was no substantial hope of a grant either from the Treasury or from the Corporation. He believed that the proposal to scrap the existing hospitals and to build two great infirmaries, one upon the north and the second upon the south side of the city, was impracticable at present. The property and income of the present hospitals would be inadequate to maintain them. A charge per patient was suggested, and he knew that patients were willing to pay, at any rate a little. He believed the only solution was to extend the plan of the docker's subscription of twopence per week to the employees in other industries. Members of the public would have to choose between paying weekly while in hospital, or paying weekly while they were well. If the latter plan were adopted through their insurance societies, these, in consideration of weekly payments, would pay the fees of those who became patients.

### THE LATEST EFFORT AT CARDIFF.

Mr. G. F. Forsdike, Lord Mayor of Cardiff, is about to start a great effort to find £150,000 for King Edward VII. Hospital, Cardiff. Half of this sum is needed to pay overdrafts on the working and builders' accounts, and the rest is needed to complete and equip the present extensions, except for a sum of £50,000 to provide a home for 250 nurses. It cannot be too often insisted that £150,000 is a small sum for a city like Cardiff and, if the wealthy shipping and colliery owners would provide it, there would be an excellent chance of appealing to the workers and the public for the increased annual income required.

### THIS WEEK'S DRUG MARKET.

Business is still somewhat quiet so far as the home trade is concerned, but exceedingly busy on the export side, with the consequence that values are well maintained nearly all round. The position of quinine is practically unchanged; there is a wide difference between the price for export and the price for the home trade value being in favour of the latter department. Menthol, camphor, and Japanese peppermint oil have not fully revived since the New Year, but it is quite probable that the upward price movement will set in again. Bromides are unchanged in price, but in view of the possibility of increased imports from Germany buyers are in doubt as to whether the present high quotations can be maintained. Lemon oil is dearer. Citric acid is rather lower in price.

